# Consensus on Prostate Cancer Treatment of Localized Disease With Very Low, Low, and Intermediate Risk: A Report From the First Prostate Cancer Consensus Conference for Developing Countries (PCCCDC)

**DOI:** 10.1200/GO.20.00515

**Published:** 2021-04-15

**Authors:** Murilo de Almeida Luz, Gustavo Cardoso Guimarães, Aguinaldo César Nardi, Alexandre Saad Feres Lima Pompeo, Álvaro Sadek Sarkis, Amr Nowier, Antônio Carlos Lima Pompeo, Archimedes Nardozza Jr, Ari Adamy Jr, Arie Carneiro, Bernardo Peres Salvajoli, Bruno Santos Benigno, Celso Heitor de Freitas Jr, Clarissa Angotti Daher Cezar Chade, Daniel Moore Freitas Palhares, Danilo Armando Citarella Otero, Deusdedit Cortêz Vieira da Silva Neto, Eduardo Franco Carvalhal, Erlon Gil, Fernando Freire de Arruda, Fernando Korkes, Gustavo Caserta lemos, Gustavo Franco Carvalhal, Ícaro Thiago de Carvalho, Ivan Federico Pinto Gimpel, José Luis Chambô, José Pontes Jr, Leopoldo Alves Ribeiro Filho, Lucas Mendes Nogueira, Marcelo Langer Wroclawski, Marcelo Roberto Pereira Freitas, Marco Antônio Arap, Marcus Vinícius Sadi, Muhammad Bulbul, Rafael Ferreira Coelho, Rafael Gadia, Raja B. Khauli, Rodolfo Borges dos Reis, Rodrigo Antônio Ledezma Rojas, Roger Guilherme Guimarães, Saad Aldousari, Robson Ferrigno

**Affiliations:** ^1^ Hospital Erasto Gaertner, Curitiba, Brazil; ^2^ A Beneficiência Portuguesa de São Paulo, São Paulo, Brazil; ^3^ Integra Urologia, Bauru, São Paulo; ^4^ Faculdade de Medicina do ABC, Santo André, Brazil; ^5^ Instituto do Câncer do Estado de São Paulo, São Paulo, Brazil; ^6^ Heliopolis, Cairo, Egypt; ^7^ Universidade Federal de São Paulo, São Paulo, Brazil; ^8^ Hospital Santa Cruz, Curitiba, Brazil; ^9^ Hospital Israelita Albert Einstein, São Paulo, Brazil; ^10^ Hospital Alemão Oswaldo Cruz, São Paulo, Brazil; ^11^ Hospital Sírio-Libanês, Brasília, Brazil; ^12^ Fundación Clinica Shaio, Bogotá, Colômbia; ^13^ Faculdade de Ciências Médicas da Santa Casa de São Paulo, São Paulo, Brazil; ^14^ Hospital Moinhos de Vento, Porto Alegre, Brazil; ^15^ Hospital Sírio-Libanês, São Paulo, Brazil; ^16^ Pontifícia Universidade Católica do Rio Grande do Sul, Porto Alegre, Brazil; ^17^ Clinica Santa Maria, Providencia, Chile; ^18^ Universidade Federal de Minas Gerais, Belo Horizonte, Brazil; ^19^ Centro Especializado de Oncologia de Florianópolis, Florianópolis, Brazil; ^20^ American University of Beirut, Beirut, Lebanon; ^21^ Universidade de São Paulo, Ribeirão Preto, Brazil; ^22^ Universidad de Chile, Santiago, Chile; ^23^ Kuwait University, Kuwait City, Kuwait

## Abstract

**PURPOSE:**

A group of international urology and medical oncology experts developed and completed a survey on prostate cancer (PCa) in developing countries. The results are reviewed and summarized, and recommendations on consensus statements for very low-, low-, and intermediate-risk PCa focused on developing countries were developed.

**METHODS:**

A panel of experts developed more than 300 survey questions of which 66 questions concern the principal areas of interest of this paper: very low, low, and intermediate risk of PCa in developing countries. A larger panel of 99 international multidisciplinary cancer experts voted on these questions to create the recommendations for treatment and follow-up for very low-, low-, and intermediate-risk PCa in areas of limited resources discussed in this manuscript.

**RESULTS:**

The panel voted publicly but anonymously on the predefined questions. Each question was deemed consensus if 75% or more of the full panel had selected a particular answer. These answers are based on panelist opinion not a literature review or meta-analysis. For questions that refer to an area of limited resources, the recommendations consider cost-effectiveness and the possible therapies with easier and greater access. Each question had five to seven relevant answers including two nonanswers. The results were tabulated in real time.

**CONCLUSION:**

The voting results and recommendations presented in this document can be used by physicians to support management for very low, low, and intermediate risk of PCa in areas of limited resources. Individual clinical decision making should be supported by available data; however, as guidelines for treatment for very low, low, and intermediate risk of PCa in developing countries have not been developed, this document will serve as a point of reference when confronted with this disease.

## INTRODUCTION

Prostate cancer (PCa) is a common cancer that affects men. The incidence of the disease has been increasing globally in recent years. Global Cancer Observatory: Cancer Today data showed that PCa was the second most frequently diagnosed cancer and the fifth leading cause of cancer mortality among men worldwide in 2012. Over the last 20 years, an increasing trend has been observed in the new cases and deaths from different cancers worldwide, especially in low- and middle-income countries.^1^ In the Americas, PCa is the most common cancer in males, with approximately 413,000 new cases and 85,000 deaths each year.^2^

**CONTEXT**

**Key Objective**
To generate a consensus on critical issues relevant to very low-, low-, and intermediate-risk prostate cancer (PCa) focused on developing countries.
**Knowledge Generated**
The panel reached consensus in recommending active surveillance for otherwise healthy patients with very low-risk PCa. For patients with a life expectancy of > 10-15 years with the diagnosis of intermediate-risk PCa, a PSA level of < 20 ng/mL, and disease confined to the prostate, the panel reached consensus in recommending radical prostatectomy. The panel recommends PSA measurement every 3-6 months for 5 years and then every year for follow-up of patients with PCa.
**Relevance**
The voting results presented in this document can be used to support the treatment of localized PCa in areas of limited resources lacking specific guidelines.

PCa can be managed with curative intent when it is still localized and can respond to treatments even in metastatic cases. Tumor growth rates vary by type of tumor, stage, and response to therapy. A US study showed that 5-year survival of localized and locally advanced tumors was 100% and the metastasis rate was 28.7%.^3^ However, when screening for PCa, one of the goals is to identify localized high-risk PCa that can be successfully treated, thereby decreasing complications associated with advanced or metastatic PCa.^4^

Although prostate-specific antigen (PSA) levels historically correlate with the presence of PCa, this test provides little information regarding disease location and extent of cancer and has both potential benefits and harms. Screening with PSA leads to overdiagnosis. Treatment of these men often results in adverse events including erectile dysfunction, urinary incontinence, and bowel symptoms, therefore providing no benefit. Follow-up of large randomized trials suggests that 20%-50% of men diagnosed with PCa through screening may be overdiagnosed.^5^ In men with 70 years and older, overdiagnosis increases rates, related to competing causes of death. The US Preventive Services Task Force does not recommend screening for PCa. After informed and understanding the risks, the preference of the patient could be evaluated.^4^

Although early detection of PCa by PSA screening remains controversial, changes in PSA threshold, frequency of screening, and addition of other biomarkers have potential to minimize overdiagnosis associated with PSA screening.^6^

In managing PCa, the first step is determining whether treatment is necessary. PCa, especially low-grade tumors, has slow growth rates and often does not require treatment, particularly in older patients with comorbidities that will limit the life expectancy to 10 additional years or less.^7^ Active surveillance (AS) is recommended for low-risk cases.

AS is based on the premise that a patient population exists that may not benefit from primary treatment of their PCa and has two goals: (1) to provide definitive treatment for men with localized cancers that are likely to progress and (2) to reduce the risk of treatment‐related complications for men with cancers that are not likely to progress. Conceptually, this form of treatment was developed because of concerns about overtreatment and overdiagnosis of PCa given that patients diagnosed with PCa are more likely to die of non-PCa causes and may be unnecessarily exposed to treatment‐related morbidity with limited long‐term survival advantage.^8^ For men with low-risk disease and favorable type of intermediate-risk disease, AS may be the best strategy for a noninvasive management protocol.^9^

## TREATMENT: LOCALIZED LOW-RISK (AND VERY LOW-RISK) PCa

AS is a treatment approach that allows men with low-risk PCa to waive surgery or radiation, monitoring their cancer. AS usually includes regular, repeated PSA testing and often repeated digital rectal examination and prostate biopsy. Definitive treatment with surgery or radiation therapy is offered to cases where the cancer evolves. Watchful waiting is another strategy for patients with low-risk PCa. In the United States over the past several years, AS has become a common choice among professionals.^4^

Low-risk PCa and many cases of the favorable subtype of intermediate risk are indolent, with almost no metastatic potential. Multiple studies identified these patients and encourage the use of conservative management in these patients. An important part of this conservative management protocol is to identify about 30% of patients who have low-risk disease but actually harbor higher-risk disease (missed by the initial random biopsy). There is now broad medical consensus that men with lower-risk disease can defer treatment. Studies of AS with selective intervention for patients who are reclassified as higher risk over time (using repeat biopsy, imaging scans, or biomarker results) have shown that the approach is safe in the intermediate- to long-term, with a 3% cancer specific mortality at 10-15 years.^9^

The panel reached consensus in recommending AS for treatment for an otherwise healthy patient diagnosed with very low-risk PCa (92.94%), including in an area of limited resources and with no magnetic resonance imaging (MRI) evaluation available (84.88%).

For an otherwise healthy patient diagnosed with low-risk PCa, most of the panel (57.65%) selected AS as the recommended treatment, although some (17.75%) selected all options that include AS, radical prostatectomy (open/LAP/or robotic approach available), focal therapy (if there is an index lesion on MRI), external beam radiation (preferred intensity-modulated radiotherapy [IMRT]), brachytherapy, and external beam radiation (preferred IMRT) with or without brachytherapy plus androgen deprivation therapy (ADT). For the same low-risk patient, in an area with limited resources, most of the panel (68.60%) recommended AS for treatment, and one quarter (24.42%) chose radical prostatectomy (only open approach is available).

When AS is selected for the treatment of very low- and low-risk PCa, the panel was split in recommending the timing for which at least one confirmatory biopsy must be performed for a patient to be considered for AS protocol, with almost half recommending between 6 and 12 months (48.84%) and some (38.37%) recommending 12-24 months. In the same case, in an area with limited resources, the panel reversed its recommendation with 49.41% selecting 12-24 months and 35.29% indicating 6-12 months. For follow-up tests, half of the panel (50%) recommended annual MRI and digital rectal exam (DRE) and PSA measurement every 6 months and biopsy in the case of signs of progression, and some (40.48%) made the same recommendation with difference of the biopsy every 1-1.5 years. When considering an area with limited resources and no MRI available, for follow-up tests, most of the panel (55.29%) recommended DRE and PSA measurement every 6 months and biopsy every 1-1.5 and one fifth (21.18%) selected DRE and PSA measurement every 12 months and biopsy every 1-1.5 years.

When recommending the timing of re-biopsy in patients on AS, most of the panel chose every 12-18 months as the first time and afterward, only in the case of signs of progression (image studies, PSA rise, or digital rectal exam) for patients with PCa, including those in an area with limited resources (61.63% and 61.18%, respectively). Some (19.77%) selected every 12-18 months on a regular basis for patients with PCa, whereas others (21.18%) recommended only in the case of signs of progression (image studies, PSA rise, or digital rectal exam) for areas of limited resources.

When considering patients with very low- or low-risk PCa, who are candidates for watchful waiting because of age, comorbidities, or an life expectancy of < 10-15 years, are asymptomatic and refuse to be followed expectantly, with the panel split in recommending external beam radiotherapy (preferred IMRT) (40.48%), surgery (21.43%), and focal therapy—if no LUTS symptoms and unilateral index lesion on MRI (14.29%) for treatment considering prostate volumes higher than 50 mm.^3^ For the same patients, in an area of limited resources, much of the panel (43.02%) recommended external beam radiotherapy, with more than one quarter (27.91%) selecting surgery. The panel was divided in its recommendation for follow-up, with 38.82% indicating no evaluation until the presence of symptoms, 21.18% recommending PSA and DRE every 6 months, and 20% choosing annual MRI and DRE and PSA measurement every 6 months and biopsy in the case of signs of progression. For follow-up in areas of limited resources, most of the panel (70.59%) recommended no evaluation until the presence of symptoms.

For patients with a life expectancy of > 10-15 years, with very low- or low-risk PCa, who declined AS or who had disease progression on AS, most of the panel (72.62%) recommended radical prostatectomy for treatment. For the same patients, in institutions where there is no availability of IMRT technique, robotic and/or laparoscopic surgery nor focal therapy, consensus was reached (83.53%) in recommending radical prostatectomy (open only).

In institutions where there is no conformal external beam radiotherapy availability of IMRT technique, robotic and/or laparoscopic surgery nor focal therapy or brachytherapy, for patients with a life expectancy of > 10-15 years, with low-risk PCa, who declined AS or who had disease progression on AS, all panelists (100%) agreed on treatment with radical prostatectomy (open only).

In institutions where only conventional radiotherapy is available, most of the panelists (70.24%) agreed that most patients with PCa can be treated with external radiotherapy, while one fifth (21.43%) indicated this treatment for a minority of patients. Where there is only cobalt radiotherapy, the panelists reached consensus (80.49%) that these patients with PCa cannot be treated with external radiotherapy.

When some form of radiation is the treatment of low-risk PCa, the panel reached consensus (91.46%) in indicating that no ADT would be recommended for patients with PCa in best practice (91.46%) or areas of limited resources (97.62%).

When the treatment for low-risk PCa is radical prostatectomy, the panel reached consensus (75.58%) in agreeing that there would be no pelvic lymph node dissection, including in areas of limited resources for which the panel also reached consensus (92.77%) for the same response.

When the treatment for very low- or low-risk PCa is radical prostatectomy, consensus was reached (82.35%) in recommending that robotic surgery should be used. When considering an area of limited resources, consensus was also reached (83.13%) in recommending open surgery as the treatment of choice.

When asked if moderate hypofractionated external beam radiotherapy can be employed for very low- and low-risk PCa in institutions with no availability of IMRT and IGRT, most panelists (56.10%) agreed that it may be employed in the majority or patients; however, one third (32.93%) said it should not.

## FOLLOW-UP: VERY LOW- AND LOW-RISK PCa

After surgery, or radiotherapy (any form), with curative intent, in a patient with very low- or low-risk PCa, most panelists recommend following-up with PSA measurement every 3-6 months for 5 years and then every year (70.56% for very low risk and 73.17% for low risk), including in areas of limited resources (65.48% for very low risk and 68.75% for low risk). For the same patient, after a definitive therapy with curative intent, almost all panelists indicate that they never order imaging routinely as a follow-up for patients with PCa (92.86%), including in areas of limited resources (98.78%).

## TREATMENT: LOCALIZED INTERMEDIATE-RISK PCa

Intermediate-risk PCa represents the largest of the risk groups and comprises a heterogeneous population of patients with variable prognoses, presenting a challenge to developing standardized treatment recommendations.^10^ Each patient with PCa is assigned two grades that make up a Gleason score. A primary grade describes the cells that compose the largest area of the tumor, and a secondary grade describes the cells of the next largest area. A score of seven suggests an intermediate risk for aggressive cancer and that the primary score is a three or four.

Tumors with a primary score of three and a secondary score of four have a fairly good outlook, whereas cancers with a primary Gleason Score of four and a secondary score of three are more likely to grow and spread.^11^ Based on their clinical characteristics, patients with intermediate-risk PCa are categorized into favorable and unfavorable subgroups. Favorable patients are those who meet all of the following: (1) only one intermediate-risk factor (based on the National Comprehensive Cancer Network classification scheme), (2) a Gleason score of 3 + 4 = 7 or less, and (3) less than 50% of biopsy cores positive for cancer.^10^

Literature provides evidence that men with favorable intermediate-risk PCa have PCa-specific mortality and all-cause mortality rates similar to patients with low-risk PCa and thus may be candidates for AS, dose-escalated radiation therapy without short-term ADT, or, interestingly, standard-dose radiation therapy plus short-term ADT.^10^

The use of AS for selected, lower-risk, intermediate-grade PCa (Gleason 3 + 4 = 7 with a PSA level < 10) could be reasonable in carefully selected cases. Genomic testing may estimate and clarify the adequate relative risk of tumor progression and aggressiveness in these controversial situations.^7^ Prostate MRI can be used to follow these patients and avoids the discomfort of repeated biopsies. Close observation aims to identify patients who will significantly increase PSA levels or have clinical progress. This indicates that patients develop more aggressive cancer for whom definitive treatment should be considered.^7^

The panel reached consensus in recommending radical prostatectomy in various situations for all the survey questions for patients with a life expectancy of > 10-15 years with the diagnosis of intermediate-risk PCa, a PSA level of < 20 ng/mL, and disease confined to the prostate. Additionally, when various treatments were selected, their recommendations varied. Both are noted in Table 1.

**TABLE 1:**
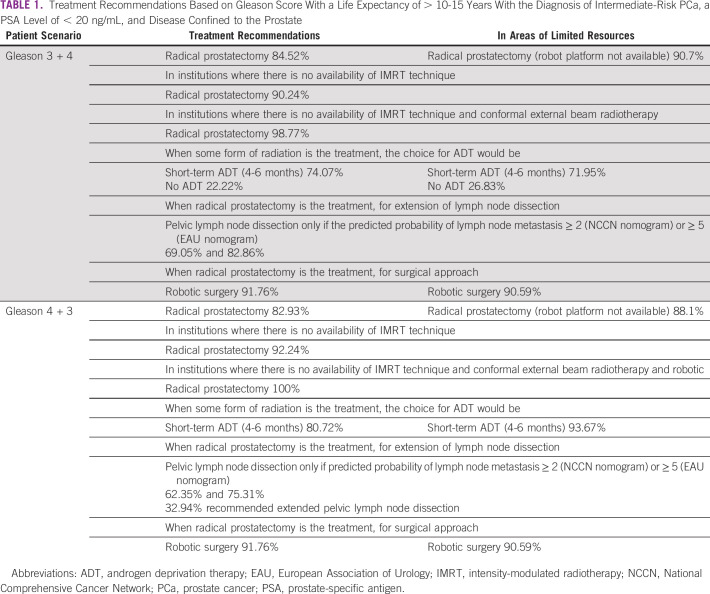
Treatment Recommendations Based on Gleason Score With a Life Expectancy of > 10-15 Years With the Diagnosis of Intermediate-Risk PCa, a PSA Level of < 20 ng/mL, and Disease Confined to the Prostate

In institutions where there is only conventional radiotherapy technique, most panelists (72.84%) agreed that the majority of patients with intermediate-risk localized PCa can be treated with external radiotherapy, whereas some panelists (17.75%) said that the minority of patients can receive this treatment. In institutions where there is only cobalt radiotherapy technique, the panel reached consensus (83.33%) in recommending that patients with intermediate-risk localized PCa cannot be treated with external radiotherapy.

When considering patients with a life expectancy of < 10-15 years, the diagnosis of intermediate-risk PCa, a PSA level of < 20 ng/mL, and disease confined to the prostate, in different situations, the panel recommendations for treatment varied, as noted in Table 2.

**TABLE 2:**
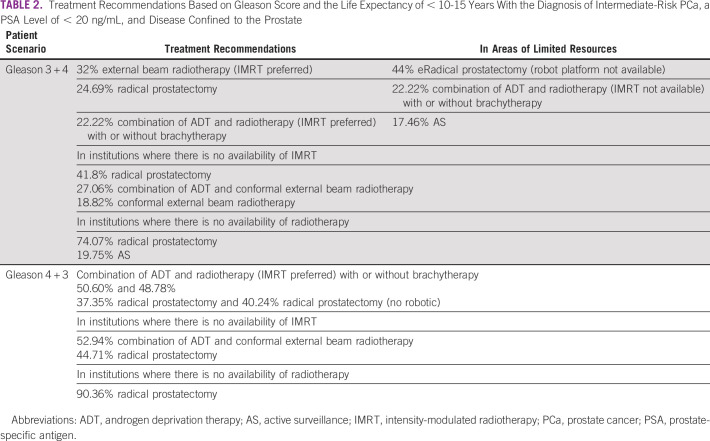
Treatment Recommendations Based on Gleason Score and the Life Expectancy of < 10-15 Years With the Diagnosis of Intermediate-Risk PCa, a PSA Level of < 20 ng/mL, and Disease Confined to the Prostate

## FOLLOW-UP: INTERMEDIATE-RISK PCa

Regarding follow-up for patients with intermediate-risk PCa, after surgery with curative intent, the panel reached consensus in recommending PSA measurement every 3-6 months for 5 years and then every year for the majority of patients (90.36%), including in areas of limited resources (93.83%).

For the same patients, after radiotherapy (any form) with curative intent (with or without ADT), the panel again reached consensus (83.75%), including in areas of limited resources (91.14%) in recommending PSA measurement every 3-6 months for 5 years and then every year for the majority of patients.

For the same patients, after a definitive therapy with curative intent, the panel reached consensus (85.19%), including in areas of limited resources (94.74%), indicating that they would never order imaging routinely as a follow-up in these patients.

In conclusion, although the panel reached consensus in recommending AS for otherwise healthy patients with very low-risk PCa, including patients in areas of limited resources, they differed in their recommendations for treatment of the low-risk patient subset.

When AS was selected as treatment for patients with very-low and low-risk PCa, including patients in areas of limited resources, there was no consensus reached on the timing for confirmatory biopsy, follow-up tests, or re-biopsy. The same is true for recommendations for treatment and follow-up for patients with very-low and low-risk PCa who are considered candidates for watchful waiting.

For patients with a life expectancy of > 10-15 years with the diagnosis of intermediate-risk PCa, a PSA level of < 20 ng/mL, and disease confined to the prostate, the panel reached consensus in recommending radical prostatectomy for patients with Gleason score 3 + 4 or 4 + 3, even in institutions where there is no availability of IMRT technique, conformal external beam radiotherapy, and robotic surgery, including in areas of limited resources.

Although PSA values provide limited information regarding the extent and location of the cancer, and in some groups of patients may have harms and benefits, it is a required test when managing patients with intermediate-risk PCa after treatment. The panel recommends PSA measurement every 3-6 months for 5 years and then every year for follow-up of patients with PCa, including in areas of limited resources where the issue may not be the availability of the test itself but the accessibility to a stable healthcare provider, a complete medical record, or the patient’s adherence to the appointments.

The recommendations from this panel in treating patients with very low-, low-, and intermediate-risk PCa in areas of limited resources are the same as or very similar to those made in developing countries for patients with a life expectancy of > 5-10 years. Differences in treatment recommendations are more apparent for patients with a life expectancy of < 5-10 years.

## References

[1] Adeloye D, David RA, Aderemi AV (2016). An estimate of the incidence of prostate cancer in Africa: A systematic review and meta-analysis. PLoS One.

[2] The Pan-American Health Organization (PAHO) (2017). Regional Experts Discuss Approaches for Prostate Cancer Screening and Early Detection in the Americas.

[3] Society AC (2013). Cancer Facts and Figures 2013.

[4] U.S. Preventive Services Task Force (USPSTF) (2018). Final Recommendation Statement: Prostate Cancer: Screening.

[5] Fenton JJ Weyrich MS Durbin S et al Prostate-specific antigen–based screening for prostate cancer: Evidence report and systematic review for the US Preventive Services Task Force JAMA 319 1914–1931 2018 29801018 10.1001/jama.2018.3712

[6] Cuzick J Thorat MA Andriole G et al Prevention and early detection of prostate cancer Lancet Oncol 15 e484–92 2014 25281467 10.1016/S1470-2045(14)70211-6 PMC4203149

[7] Leslie SW, Soon-Sutton TL, Sajjad H (2019). Prostate Cancer.

[8] Dahabreh IJ Chung M Balk EM et al Active surveillance in men with localized prostate cancer: A systematic review Ann Intern Med 156 582–590 2012 22351515 10.7326/0003-4819-156-8-201204170-00397

[9] Klotz L (2017). An Update on Active Surveillance.

[10] (2020). Favorable vs Unfavorable Intermediate-Risk Prostate Cancer: A Review of the New Classification System and its Impact on Treatment Recommendations.

[11] Martin K (2020). Gleason Score—Prostate Conditions.

